# Partial Mitochondrial Complex I Inhibitors as a Therapy for Alzheimer Disease

**DOI:** 10.1002/alz70859_105252

**Published:** 2025-12-26

**Authors:** Sergey A Trushin, Mark Ostroot, Eugenia Trushina

**Affiliations:** ^1^ Department of Neurology, Mayo Clinic, Rochester, MN USA; ^2^ Mayo Clinic, Rochester, MN USA; ^3^ Department of Molecular Pharmacology and Experimental Therapeutics, Mayo Clinic, Rochester, MN USA

## Abstract

**Background:**

Despite recent approval of monoclonal antibodies that reduce amyloid (Ab) accumulation, the development of disease‐modifying strategies targeting the underlying mechanisms of Alzheimer’s Disease (AD) are urgently needed. We identified mitochondrial complex I (mtCI) as a small‐molecule druggable target for AD. Using cryo‐EM, we previously demonstrated that the neuroprotective tool compound CP2 directly binds to the Q‐site of mtCI and weakly inhibits its activity.

**Method:**

Rational design and extensive structure‐activity relationship studies using CP2 as a tool compound allowed the development of novel classes of partial mtCI inhibitors with high selectivity, specificity, safety, and improved drug‐like properties. Target engagement, selectivity, efficacy, safety and the direct binding to mtCI were demonstrated using multiple in vitro assays. Efficacy in vivo was established using APP/PS1 mice. The best new leads, C458 and C273, were evaluated as preclinical candidates.

**Result:**

Like CP2, C458 and C273 weakly inhibit mtCI, leading to AMPK activation, which triggers a neuroprotective stress response. This response enhances oxidative stress protection, promotes autophagy, and increases mitochondrial biogenesis and turnover, ultimately protecting against Aβ toxicity. Pretreatment with the irreversible mtCI inhibitor rotenone (at non‐toxic concentrations) blocked C458 and C273 from binding to mtCI and abrogated their protection against Aβ toxicity. Both C458 and C273 exhibited excellent bioavailability, blood–brain barrier penetrance, and a clean safety profile in the CEREP 44 safety panel. Chronic administration of C458 (25 mg/kg/day in drinking water *ad lib*) to APP/PS1 mice for 7 months preserved cognition, improved long‐term potentiation, reduced oxidative stress and inflammation, and enhanced mitochondrial biogenesis. Similarly, C273 (20, 50, or 80 mg/kg/day) administered to C57BL/6 mice for one month showed no detectable side effects or toxicity in the heart or liver.

**Conclusion:**

Mechanistic studies revealed that C458 and C273 activate a mitochondrial stress response, augmenting mitochondrial biogenesis, antioxidant signaling, and autophagy, which collectively provided protection against Aβ toxicity in multiple in vitro and in vivo models of AD. These findings demonstrate that weak mtCI inhibition by novel compounds C458 and C273, with improved drug‐like properties, is neuroprotective. This supports the feasibility of developing safe and efficacious mtCI inhibitors as a disease‐modifying strategy for AD.

